# Targeting the ubiquitin-proteasome system: a novel therapeutic strategy for neuroblastoma

**DOI:** 10.3389/fonc.2024.1443256

**Published:** 2024-09-26

**Authors:** Yangshen He, Jianing Wang, Tiantian Xiao

**Affiliations:** ^1^ Department of General Medicine, Dongguan Hospital of Integrated Chinese and Western Medicine, Dongguan, China; ^2^ The First Affiliated Hospital of Jinan University, Guangzhou, China

**Keywords:** neuroblastoma, ubiquitination, targeted therapy, cancer therapy, proteasome inhibition

## Abstract

Neuroblastoma (NB) stands as a common and formidable malignant tumor among children, characterized by marked tumor heterogeneity and resistance to conventional treatments. Central to the regulation of protein stability, localization, and function is the process of ubiquitination—a critical protein modification. The therapeutic potential of drugs that target deubiquitination, demonstrated in the treatment of refractory multiple myeloma, warrants investigation in the context of NB. This review endeavors to demystify the intricate biological implications of ubiquitination within NB pathology, synthesize the current landscape of preclinical studies focused on the inhibition of the ubiquitin-proteasome system in NB, and assess the viability of this strategy as an innovative therapeutic frontier.

## 1 Introduction

Neuroblastoma (NB) is a malignant tumor originating from neural crest cells and predominantly affects children under 5 years old (1, 2). Accounting for 6%–10% of childhood cancers, NB ranks as the most common extracranial solid tumor in children (3). The heterogeneous pathology and clinical presentations of NB complicate treatment and make prognosis assessment challenging. Current therapeutic approaches encompass surgery, chemotherapy, radiotherapy, and hematopoietic stem cell transplantation. Whereas low-risk patients exhibit a more favorable prognosis, high-risk patients face a notably low 5-year survival rate (4). Although the ganglioside (GD2) monoclonal antibody shows promise in improving NB prognosis, its clinical utility is hindered by severe neuropathic pain and antigen remodeling mechanisms (5). Children's Oncology Group (COG) organized a study called ABNL1221 to explore the effectiveness of the I/T/DIN chemotherapeutic immune-regimen of GD2 monoclonal antibodies combined with irinotecan and temozolomide in the treatment of high-risk recurrent NB. The results of the two studies indicate that the objective response rate of immunochemotherapy in the treatment of highly refractory recurrent NB is only 41.5%–53% (6, 7). Another study from the Barcelona Pediatric Cancer Center explored the efficacy and safety of GD2 monoclonal combined with irinotecan and temozolomide in another regimen called Hu3F8 (H), irinotecan (I), temozolomide (T), and sargramostim (S) (HITS) for the treatment of high-risk recurrent NB. Although the CR rate reached 47% in the group with the best response, the rate of adverse reactions of grade 3 toxicity or greater was 85% (8). An additional series of studies have attempted to further activate the Antibody-Dependent Cell-Mediated Cytotoxicity (ADCC) effect of NK cells with the addition of Interleukin-2 (IL-2) and IL-15 cytokines while using GD2 monoclonal antibodies, but differences have been reported between the results. The successful use of GD2 mab in high-risk recurrent NB has improved response rates and prognosis, but only about 50% of the population has benefited. In a pioneering study, third-generation GD2-directed Chimeric Antigen Receptor T-Cell (CAR-T) cell therapy has been administered to individuals with metastatic melanoma and solid tumors like colorectal cancer for the first time, demonstrating its viability, safety, and immune response, but with modest clinical benefits (ACTRN12613000198729). Consequently, researchers are exploring novel treatment strategies to enhance outcomes for high-risk patients with NB (9).

Ubiquitination modification, a crucial post-translational protein modification process, involves the covalent attachment of ubiquitin molecules to target proteins to regulate their stability, activity, and intracellular localization (10). Ubiquitination begins when an enzyme called ubiquitin-activating enzyme (E1) turns on ubiquitin molecules. These molecules are then passed to another enzyme known as ubiquitin-conjugating enzyme (E2). After that, the E2 enzyme links up with a specific enzyme called ubiquitin ligase (E3), which can identify a unique protein that it is looking for. The E3 enzyme helps attach the ubiquitin to a specific spot on this protein, which is a lysine amino acid (10). This process can be iterated to form ubiquitin chains that label substrate proteins for proteasome-mediated degradation or other ubiquitination-dependent regulatory mechanisms (10). Ubiquitination modification has garnered significant attention in the realm of tumor biology, with studies demonstrating its impact on the stability and activity of various tumor suppressors and oncogenes (11, 12).

P53 stands as a preeminent tumor suppressor, central to the mouse double minute 2 homolog (MDM2)/mouse double minute X homolog (MDMX)–p53–negative feedback loop, which includes MDM2 and MDMX proteins (13). This regulatory circuitry is instrumental in modulating essential biological processes, including cancer cell proliferation, growth, cell cycle regulation, apoptosis, senescence, angiogenesis, and immune responses (14). The stability of P53, MDM2, and MDMX is subject to precise control by the ubiquitin-proteasome system (15). In particular, inhibitors of the ubiquitin-specific protease 7 (USP7) have been shown to enhance the degradation of MDM2 and MDMX, liberating and activating p53 to exert its tumor-suppressive functions (15). Ubiquitination also plays a role in governing a plethora of key signaling cascades, such as the phosphoinositide 3-kinase (PI3K)/AKT/mammalian target of rapamycin (mTOR), Nuclear Factor E2-related factor 2 (NRF2), and Hypoxia-inducible factors (HIF) pathways (16–18). Disruptions in ubiquitination can lead to excessive activation of these pathways, fostering the proliferation, differentiation, metabolic activities, and survival of tumor cells (15). Specific E3 ubiquitin ligases contribute to cell cycle dysregulation and unchecked tumor proliferation by promoting the degradation of cell cycle inhibitors (19). In light of ubiquitination’s pivotal function in cancer development and progression, a host of E3 ubiquitin ligases and deubiquitinating enzymes (DUBs) have been identified as promising targets for anti-cancer therapy (12). Research is focused not only on direct inhibitors of enzymes related to ubiquitination but also on enhancing the efficacy of current treatments through the manipulation of ubiquitination processes. For example, bortezomib, a proteasome inhibitor, has been extensively utilized in the management of multiple myeloma (MM) with considerable success (20). The efficacy of proteasome inhibitors in MM treatment underscores the potential of targeting the proteasomal degradation, the final stage of ubiquitination, as a viable therapeutic strategy (20). A phase I study of bortezomib in combination with irinotecan in patients with relapsed/refractory high-risk NB has shown initial effectiveness (21).

Deubiquitination is the opposite of ubiquitination. In this process, enzymes known as DUBs remove ubiquitin labels from proteins. This action stops the proteins from being broken down by a cell’s waste disposal system called the proteasome (22). Targeted inhibition or removal of ubiquitination enzymes can specifically break down proteins of interest. This approach might offer a way to affect proteins that are hard to target with drugs, like some transcription factors (for example, MYCN), enzymes that cause resistance to treatments, and proteins engaged in complicated interactions between different proteins, which usually cannot be addressed by small-molecule drugs (22, 23). Of course, there are two strategies including hydrophobic tagging (HyT) and proteolysis-targeting chimera (PROTAC) have been developed for degrading a broader range of proteins (24, 25). The mostly used hydrophobic moieties include adamantine and BOC3-Arg. HyT had limited applications, because BOC3-Arg was found to inhibit the mammalian target of rapamycin complex 1 (mTORC1) pathway (26). A PROTAC molecule consists of a ligand (mostly small-molecule inhibitor) of the protein of interest (POI) and a ligand of an E3 ubiquitin ligase, upon binding to POI, the PROTAC can recruit E3 for proximity-induced ubiquitination of POI, which is then subjected to degradation by endogenous 26S proteasome (26).

This approach is particularly promising for the treatment of cancers. Delving into the precise mechanisms by which ubiquitination operates in NB is set to enhance our understanding of the tumor’s biology and may unveil new therapeutic targets. This review will thoroughly explore the role of ubiquitination in the etiology, progression, and therapeutic strategies for NB, assessing its viability as a therapeutic target. It is our aspiration that these scholarly endeavors will pave the way for more efficacious and tailored treatment regimens for patients with NB, consequently ameliorating their clinical outcomes and overall quality of life.

## 2 The dynamic equilibrium of ubiquitination and deubiquitination in NB pathogenesis

### 2.1 Advancing insights into high-risk mutations in NB

In the realm of NB research, a spectrum of high-frequency, high-risk genetic alterations have been pinpointed, encompassing N-MYC amplification, ALK (anaplastic lymphoma kinase) dysregulation, ATRX (alpha thalassemia/mental retardation X-linked) abnormalities, and tumor protein 53 (TP53) mutations (4, 23, 27). Notably, N-MYC amplification stands out as a critical oncogenic event, tightly linked to the exacerbation of disease severity. The MYCN oncogene product, a pivotal transcription factor, sees its stability and functional activity rigorously modulated by the ubiquitination-proteasome axis (28). MYCN’s role in fueling cell cycle advancement and neuronal differentiation is particularly pronounced during embryonic development, positioning it as a compelling target for therapeutic intervention.

A cadre of E3 ubiquitin ligases—encompassing F-box and WD repeat domain containing 7 (Fbxw7), Herpesvirus-associated ubiquitin-specific protease (HAUSP), Tripartite Motif Containing 32 (Trim32), and HECT, UBA and WWE domain containing E3 ubiquitin protein ligase 1 (Huwe1)—and deubiquitinases, including USP3 and USP7, orchestrate the fine-tuned ubiquitination-deubiquitination balance of MYCN, thereby dictating its subcellular concentrations and stability (28–30). HAUSP (USP7), a deubiquitinase, is implicated in the deubiquitination of MYCN protein, thereby thwarting its proteasomal degradation (30, 31). Empirical evidence from NB cell lines LAN-1 and SK-N-BE, both characterized by MYCN amplification, supports the notion that HAUSP ablation augments MYCN protein turnover (31). An upregulated HAUSP signature correlates with an ominous prognosis in patients with NB (31). In addition, the expression of Cyclin B1 Interacting Protein 1 (CCNB1IP1) is observed to surge in NB cells with MYCN amplification (29). CCNB1IP1 engages in a competitive binding tussle with Fbxw7 for MYCN, averting its ubiquitination and subsequent proteasome-mediated degradation, culminating in the heightened stability of MYCN protein and the propagation of NB cell proliferation and tumorigenic processes (29).

The tumor suppressor p53, a sentinel regulator of the cell cycle, similarly falls under the regulatory purview of the ubiquitination machinery. The interaction between HAUSP and the p53–Mdm2 complex, leading to p53’s deubiquitination, stands as a seminal example of the role DUBs play in protein stability regulation (32). Ubiquitinated p53 is conventionally targeted for degradation by the 26S proteasome, a pivotal mechanism for intracellular p53 homeostasis (32). The P300 protein, in its capacity as a transcriptional coactivator, also wields ubiquitin ligase capabilities, further influencing p53 ubiquitination (33). The stabilization of p53 through deubiquitination, mitigating its proteasomal degradation, is instrumental in its antitumor function in NB (34). Moreover, an innovative strategy leveraging the ubiquitination-proteasome system involves harnessing USP7 inhibitors to enhance the ubiquitination of the MDM2/MDMX negative regulatory elements within the p53 regulatory loop, thereby amplifying p53’s anti-NB efficacy (30). The focus is on highlighting that this mechanism is relevant for individuals capable of producing a functional p53 protein, and it may not be applicable to patients with NB with TP53 gene mutations (30, 32). Nonetheless, investigations into the role of ubiquitination in other high-risk mutations associated with NB, beyond MYCN, remain sparse, potentially attributable to the infrequent occurrence of these alterations.

### 2.2 The impact of ubiquitination on NB cell proliferation, apoptosis, and migration

UBE4B, an E3/E4 ubiquitin ligase, has been found to be negatively correlated with epidermal growth factor receptor (EGFR) levels in NB, with the degradation rate of EGFR being closely associated with the levels of UBE4B within cells (35–37). Enhanced catalytic activity of UBE4B leads to reduced sensitivity to EGFR inhibitors (37). Depletion of UBE4B significantly inhibits NB proliferation and migration and augments the apoptotic response to the EGFR inhibitor Cetuximab (37). UBE4B can also form a complex with ITCH, promoting the Lys48/Lys63 branched ubiquitination and proteasomal degradation of Ku70 and c-FLIPL, resulting in increased apoptosis of NB cells (36). The TRIM (tripartite motif) protein family, through its E3 ubiquitin ligase activity, is involved in the regulation of various biological processes in NB, including tumor suppression and oncogenic processes (38). For instance, TRIM11 regulates neurogenesis by ubiquitinating PAX6, whereas TRIM16 promotes cell differentiation by enhancing the transcriptional activity of RARβ (38). TRIM21 facilitates cancer proliferation by ubiquitinating various cancer-promoting proteins (such as NF-κB, STAT3, BCL2, p53, p27, and Snail) and promotes their proteasomal degradation by increasing their ubiquitination (39, 40). Similarly, the E3 ligase Makorin Ring Finger Protein 2 (MKRN2) has been shown to regulate the proliferation and migration of human NB SHSY5Y cells by ubiquitinating Insulin-like Growth Factor 2 mRNA-binding Protein 3 (IGF2BP3) (41). These findings underscore the significance of UBE4B, the TRIM protein family, and MKRN2 in NB cell proliferation, apoptosis, and migration.

### 2.3 The influence of ubiquitination on NB cell differentiation

One of the hallmarks of NB is the impairment of cellular differentiation, which is critical for tumor formation. REST (repressor element-1 silencing transcription factor) is a key transcriptional repressor essential for the maintenance, self-renewal, and differentiation of NB (42). The deubiquitinase USP3 interacts with REST, stabilizing and prolonging the half-life of REST protein, thereby counteracting its ubiquitination in NB (42, 43). Silencing of USP3 reduces the self-renewal capacity of NB cells and promotes differentiation induced by retinoic acid. The absence of USP3 *in vitro* and *in vivo* models has been shown to decrease NB cell proliferation, migration, and invasion and inhibit tumor growth (43). The SCFβ– β-transducin repeat-containing protein (TRCP) (Skp1/Cullin/F-box protein β–TRCP) complex also regulates neuroblast differentiation by degrading REST protein, which is one of the mechanisms by which retinoic acid promotes NB differentiation (44). The accumulation and impaired degradation of inhibitory differentiation proteins represent another factor hindering the differentiation of NB cells. Xuan Li et al. discovered that calcium/calmodulin-dependent protein kinase II can promote the autophagy of inhibitory differentiation proteins through the ubiquitination pathway, thereby facilitating NB cell differentiation (45). Cylindromatosis (CYLD), another deubiquitinase, is associated with tumorigenesis due to the loss of its activity and regulates the NF-κB signaling pathway by removing Lys63 and linear-linked ubiquitin chains from substrates (46). Retinoic acid–mediated NB differentiation restores CYLD expression and promotes CYLD SUMOylation (46). This post-translational modification inhibits CYLD’s deubiquitination activity toward TRAF2 and TRAF6, activating the NF-κB signaling pathway. Overexpression of non-SUMOylated CYLD mutants in NB cells reduces retinoic acid–induced NF-κB activation and cell differentiation (46).

## 3 Preclinical insights into deubiquitination in NB

Inhibiting the ubiquitin-proteasome system to manipulate the degradation of targeted substrate proteins represents a pivotal approach in therapeutic discovery. Human cells harbor a diverse array of over 100 deubiquitinases, categorized into seven distinct families based on structural attributes, including ubiquitin-specific proteases (USPs), ubiquitin C-terminal hydrolases (UCHs), and ovarian tumor (OTU) proteases (47). The USP family stands out for its comprehensive characterization in structural and functional studies, as well as for the abundance of literature on its small-molecule modulators.

USP5, a deubiquitinase enzyme, plays a role in regulating the binding between E3 ubiquitin ligases and their substrate proteins. SE486-11 has been crafted as an inhibitor of USP5 for NB therapeutics (48). This compound exhibits potent activity against NB cells (IC50 ≤ 2 µM) and demonstrates minimal cytotoxicity to normal cells (IC50 ≥ 15 µM). The mechanism by which SE486-11 operates involves modulating USP5 to enhance MYCN ubiquitination while also potentiating the effects of histone deacetylase (HDAC) inhibitors (48). Formononetin, a natural USP5 inhibitor identified in plants such as Trifolium pratense, is known for its array of physiological effects, including anti-neurodegenerative properties (49). It prompts cell death in the neuronal cell line SH-SY5Y by engaging the PI3K/Akt-Nrf2 antioxidant gene pathway and inhibiting MAPK-mediated apoptosis (49). Suramin, another recognized USP5 inhibitor, exerts cytostatic effects at concentrations ranging from 50 to 400 µg/mL in SH-SY5Y cells (50). Flow cytometric analysis of cells treated with Suramin indicates a halt in the G1/G0 phase of the cell cycle (50). Nevertheless, a conflicting study has shown that Suramin at 100 µM concentration effectively shields cerebellar neurons and NB-2a cells from apoptosis, as evidenced by the suppression of caspase 3–like activity in NB-2a cells and the prevention of both high molecular weight (HMW) and internucleosomal DNA fragmentation (51). This discrepancy raises the intriguing possibility that varying concentrations and doses of USP inhibitors could exert differential biological responses in identical tumor cell lines.

USP7 (also known as HAUSP) was identified as a regulator of MYCN function in NB (31). HAUSP interacts with MYCN protein, inducing its deubiquitination and subsequent stabilization (31). Conversely, RNA interference–mediated knockdown of USP7 in NB cancer cell lines or genetic ablation of USP7 in mouse models disrupts MYCN stability, thereby inhibiting its function. Notably, HAUSP is more abundant in patients with NB with poorer prognosis, and its expression is significantly correlated with MYCN transcriptional activity. Additionally, small-molecule inhibitors of USP7 have been shown to significantly inhibit the growth of human NB cells with MYCN amplification in xenograft mouse models (31).

P22077, a representative small-molecule inhibitor of USP7, has been validated for its ability to induce MYCN protein degradation and inhibit NB cell growth (31). Its effects are particularly pronounced in cell lines with MYCN amplification and in xenograft models (31). Interestingly, P22077 can strongly induce apoptosis in NB cells with an intact USP7–HDM2–p53 axis but not in cells with p53 mutation or lacking human MDM2 homolog (HDM2) expression (52). P22077 stabilizes p53 by promoting HDM2 protein degradation and significantly enhances the cytotoxicity of doxorubicin (Dox) and etoposide (VP-16) to NB cells (52). Furthermore, P22077 can also re-sensitize chemo-resistant NB cells (LA-N-6) to chemotherapy. Another study showed that BIRC5/Survivin inhibitor, YM155, specifically inhibits the growth of MYCN-amplified (MNA) NB cells *in vitro* and inhibits USP7 deubiquitinase activity *in vitro* using Ub-amino methyl coumarin (Ub-AMC) as a substrate (53). *In vivo* studies further demonstrated that YM155 significantly inhibited tumor growth in the MNA NB xenograft model (53). These findings suggest that USP7 inhibitors may represent a novel approach for treating high-risk NB with MYCN amplification and non-p53 mutations, both as a monotherapy and as an effective adjunct to existing chemotherapy regimens.

b-AP15, a small-molecule inhibitor targeting USP14, has been shown to induce apoptosis and inhibit cell proliferation in NB. In SH-SY5Y xenograft mouse models, b-AP15 also significantly inhibited tumor growth. When used in combination with traditional chemotherapy drugs such as doxorubicin or VP-16, b-AP15 exerts synergistic antitumor effects on NB (54). Preclinical studies are ongoing regarding the use of the USP family as a monotherapy or in combination with existing chemotherapy regimens for NB treatment. Other similar targets include USP22 (55), USP3 (56), USP29 (57), as well as small ubiquitin-like modifiers (SUMOs) or SUMOylation.

NVP-BEZ235 is a dual kinase inhibitor targeting both PI3K and mTOR kinases and has been shown to inhibit NB cell proliferation and induce cell cycle arrest at the G0/G1 phase (58, 59). One of its mechanisms may involve activating GSK3β (glycogen synthase kinase 3β) to trigger the ubiquitin-proteasome pathway degradation of cyclin D1 and E1. NVP-BEZ235 has also shown similar anti-proliferative effects in NB xenograft mouse models (59). Some researchers have found that targeting the PI3K/Akt/mTOR signaling pathway can enhance chemotherapy sensitivity in NB (60).

ARV-825 is a novel extra terminal (Bromodomain and Extra Terminal domain (BET)) inhibitor using PROTAC technology that degrades target proteins by the proteasome. Inhibition of bromodomain and BET proteins shows great potential in multiple of *Myc*-driven tumors. A study showed that ARV-825 treatment robustly induced proliferative suppression, cell cycle arrest, and apoptosis in NB cells. Moreover, ARV-825 efficiently depleted BET protein expression, subsequently repressing the expression of MYCN or c-Myc. In the NB xenograft model, ARV-825 profoundly reduced tumor growth and led to the downregulation of BRD4 and MYCN expression in mice (61).

MZ1 is a novel BET inhibitor that employs PROTAC technology for proteasomal degradation of target proteins and has shown excellent effects in some tumors. A study observed that MZ1 suppressed MYC-amplified NB cell proliferation and normal cell cycle while simultaneously boosting cell apoptosis. MZ1 also provides a significant therapeutic impact *in vivo*, which imply that MZ1 might be exploited as a possible therapeutic method for NB therapy (62).

In summary, preclinical research on NB primarily focuses on USP5, USP7, USP14, and some key signaling pathway kinase inhibitors. BET inhibitors based on PROTAC technology have been studied in a small number of NB studies, which tentatively demonstrate the possibility of future targeted therapies for MYC-driven tumors. Other USP inhibitors, including PYR-41, WP1130, Tolmidine, vialinin A, gossypetin, and some proteasome inhibitors, have shown promising results in preclinical studies of other cancers but are relatively understudied in NB (63, 64).

## 4 Clinical investigation of deubiquitinating agents: a new hope for NB

The potential of targeting the disrupted UPS in MM was first established with the introduction of the first-in-class proteasome inhibitor bortezomib. The small-molecule inhibitor VLX1570 and its analog b-AP15 specifically inhibit the activity of USP14 and Ubiquitin Carboxyl-Terminal Hydrolase L5 (UCHL5) within the 19S regulatory subunit. In a phase I clinical trial for patients with refractory/relapsed MM, VLX1570 was administered to 14 participants. Notable antitumor effects were observed at doses exceeding 0.6 mg/kg. The trial was halted due to severe lung damage and bone marrow failure in two patients, but the observed antitumor activity corroborated preclinical findings (65). Other agents under investigation include KSQ-4279, an allosteric inhibitor of USP1, and a selective inhibitor of USP30, both of which are being evaluated in phase 1 clinical trials for solid tumors and chronic kidney disease, respectively (66, 67). The first study of bortezomib in children with refractory solid tumors was ADVL0015 to evaluate the safety and efficacy of bortezomib in 15 children with refractory solid tumors. However, no objective response was observed in 11 evaluable patients (68). Another phase I study of bortezomib in combination with irinotecan in patients with relapsed/refractory high-risk NB showed that two of 17 (12%) evaluable patients showed objective responses lasting more than 40 courses, including one partial remission and one complete remission. Four patients (23%) had prolonged stable disease (SD) lasting six or more courses, with a total of 35% study patients demonstrating clinical benefit in the form of prolonged Objective reaction (OR) or SD (21).

## 5 Summary and future challenges

This review has examined the role of ubiquitination in the development, progression, and treatment of NB, underscoring its significance as a critical protein modification process that regulates the stability, localization, and function of key proteins. The article highlights the potential of drugs targeting DUBs in treating refractory MM, but their application in NB therapy remains to be clarified. By delving into the biological implications of the UPS in NB, current preclinical research, and its potential as an innovative therapeutic approach, this article aims to provide novel insights and strategies for NB treatment. Finally, we summarized the challenges and difficulties to be faced in the future:

1. Mechanistic complexity: Elucidating the intricate interactions among E3 ligases, DUBs, and their regulatory networks in NB is crucial for therapeutic development.
2. Drug precision: Creating targeted small-molecule inhibitors for NB ubiquitination enzymes is challenging to ensure selectivity and minimize toxicity.
3. Resistance management: Addressing potential resistance in NB cells by understanding how they adapt to ubiquitination inhibitors is vital.
4. Clinical trial design: Transitioning from preclinical to clinical trials requires well-planned studies to evaluate drug safety, efficacy, and optimal dosing in diverse NB patient groups.
5. Combination therapies: Investigating the synergy and side effects of combining ubiquitination inhibitors with other treatments like chemotherapy, radiotherapy, and immunotherapy for improved NB outcomes.

In conclusion, although the ubiquitin-proteasome system offers new hope for NB treatment, its successful clinical application requires overcoming a range of scientific and clinical challenges. Future research should focus on improving the selectivity and specificity of drugs, understanding resistance mechanisms, and designing effective clinical trial strategies.
